# Co-design and evaluation of a patient-centred transition programme for stroke patients, combining case management and access to an internet information platform: study protocol for a randomized controlled trial - NAVISTROKE

**DOI:** 10.1186/s12913-022-07907-5

**Published:** 2022-04-22

**Authors:** Anne Termoz, Marion Delvallée, Eléonore Damiolini, Mathilde Marchal, Marie Preau, Laure Huchon, Stéphanie Mazza, Ouazna Habchi, Estelle Bravant, Laurent Derex, Norbert Nighoghossian, Serkan Cakmak, Muriel Rabilloud, Angélique Denis, Anne-Marie Schott, Julie Haesebaert

**Affiliations:** 1 Research on Healthcare Performance (RESHAPE), Université Claude Bernard Lyon 1, INSERM U1290, Lyon, France; 2 Service Recherche et Epidémiologie Cliniques, Hospices Civils de Lyon, Pôle de Sante Publique, Lyon, France; 3 Groupe de Recherche en Psychologie Sociale (GRePS), Université Lyon 2, Lyon, France; 4 Médecine Physique et Réadaptation, Hospices Civils de Lyon, Hôpital Henry Gabrielle, Lyon, France; 5 Service Neuro-vasculaire, Hospices Civils de Lyon, Hôpital Pierre Wertheimer, Lyon, France; 6 Service Neuro-vasculaire, Hôpital Nord Ouest, Villefranche-sur-Saône, France; 7 Service de Biostatistique et Bioinformatique Hospices Civils de Lyon Pôle Santé Publique, Lyon, France; 8 Laboratoire de Biométrie et Biologie Évolutive, Université Claude Bernard Lyon 1, CNRS, UMR 5558, Équipe Biostatistique-Santé, Villeurbanne, France

**Keywords:** Stroke, Patient-centred transition programme, Hospital discharge

## Abstract

**Background:**

Stroke affects many aspects of life in stroke survivors and their family, and returning home after hospital discharge is a key step for the patient and his or her relatives. Patients and caregivers report a significant need for advice and information during this transition period. Our hypothesis is that, through a comprehensive, individualised and flexible support for patients and their caregivers, a patient-centred post-stroke hospital/home transition programme, combining an Internet information platform and telephone follow-up by a case manager, could improve patients’ level of participation and quality of life.

**Methods:**

An open parallel-group randomized trial will be conducted in two centres in France. We will recruit 170 adult patients who have had a first confirmed stroke, and were directly discharged home from the stroke unit with a modified Rankin score ≤3. Intervention content will be defined using a user-centred approach involving patients, caregivers, health-care professionals and social workers. Patients randomized to the intervention group will receive telephonic support by a trained case manager and access to an interactive Internet information platform during the 12 months following their return home. Patients randomized to the control group will receive usual care. The primary outcome is patient participation, measured by the “participation” dimension score of the Stroke Impact Scale 6 months after discharge. Secondary outcomes will include, for patients, quality of life, activation, care consumption, as well as physical, mental and social outcomes; and for caregivers, quality of life and burden. Patients will be contacted within one week after discharge, at 6 and 12 months for the outcomes collection. A process evaluation alongside the study is planned.

**Discussion:**

Our patient-centred programme will empower patients and their carers, through individualised and progressive follow-up, to find their way around the range of available healthcare and social services, to better understand them and to use them more effectively.

The action of a centralised case manager by telephone and the online platform will make it possible to disseminate this intervention to a large number of patients, over a wide area and even in cases of geographical isolation.

**Trial registration:** ClinicalTrials NCT03956160, Posted: May-2019 and Update: September-2021.

**Supplementary Information:**

The online version contains supplementary material available at 10.1186/s12913-022-07907-5.

## Background

With the improvement of acute care organisation, stroke prognosis is continually improving [1], and the number of patients returning home directly after acute stroke unit management is dramatically increasing [2]. For instance, in France, 70% of patients return home directly after treatment in a stroke centre [3], and this is a key step for the patient and his or her relatives [4]. Due to the brutality of stroke and increasingly shorter lengths of hospital stay, patients and their families experience great difficulties adapting quickly to the patient's new state of health or dependence and the new caregiver role of family members. Following the acute phase treatment, the patient’s care pathway involves several different types of health and social workers. However, the healthcare system is complex and difficult for patients and their caregivers to understand [5], and there is a lack of support and of relevant, scientifically validated, information during the transition from hospital to home; this has significant negative consequences for the patient (reduced functional prognosis, quality of life, increased risk of recurrence) and his or her caregiver (increased perceived burden, decreased quality of life, socio-economic impact) [5, 6]. Patients and caregivers report a significant need for advice and information during this transition period. They seek individualised, good quality information that changes in nature over time in line with the evolution of the needs and recovery of the patient [7, 8]; the provision of information through an Internet platform could meet these characteristics, in association with tailored support by a case manager to ensure continuity of care and improve care pathway. Existing transition programmes mainly focus on home functional rehabilitation and do not offer a comprehensive approach, responding to patients and caregivers needs that are wider [9–13]. The recently published MISTT trial conducted in the United States showed that such a programme, associating a social worker-led case management and a dedicated website, had promising results providing a significant improvement in patient-reported outcomes related to physical health [14, 15]. However, the authors acknowledged that the mechanisms of actions of the intervention remain uncertain since the website use by patients was limited and no process evaluation alongside the trial had been conducted [16]. In addition, the transferability of the programme had not been studied and sociocultural differences might hinder the generalisation of conclusions to other healthcare contexts. In France, no such program has been developed to date for stroke, this is why we aim to develop a stroke transition program in partnership with patients and families, and assess its efficacy. Our hypothesis is that, through a comprehensive, individualized and flexible support for patients and their caregivers, a patient-centered post-stroke hospital/home transition program, combining an Internet information platform and telephone follow-up by a case manager, could improve patients’ level of participation and quality of life. We present herein the protocol for the NAVISTROKE trial, a superiority randomized controlled trial evaluating the efficacy of this programme.

## Objectives

The main objective of the trial is to assess the impact of a patient-centred post-stroke transition programme, combining an Internet information platform and telephone follow-up by a case manager, on changes in the level of patient participation, evaluated by the Stroke Impact Scale (SIS), 6 months after their return home compared to usual care. The transition programme aims to empower patients and their caregivers, and help them to identify, understand and use the available medical and medico-social services according to their needs to improve their recovery. Secondary objectives are to study the efficacy of the programme on the following patient-reported outcomes: patients’ level of activation, quality of life, anxiety, depression, fatigue, and sleep quality at 6 and 12 months; on patients’ functional prognosis, attainment of targeted cardiovascular risk factor objectives, healthcare access, as well as occupational and social functioning at 12 months. We will also measure the efficacy of the programme on informal caregivers’ burden, quality of life, anxiety, depression, and occupational and social functioning. A process evaluation of the intervention implementation will be conducted using a mixed-method approach based on the Medical Research Council guidance [17, 18]. Semi-structured interviews will be conducted to understand the barriers and facilitators perceived by patients and the case manager.

## Methods/design

The reporting of the study protocol follows the SPIRIT guidelines [19], the description of the planned intervention is guided by the template for intervention description and replication (TIDieR) checklist [20]. Patient and public involvement is reported according to the GRIPP2 reporting checklist [21].

### Study design

The NAVISTROKE trial is a 2-phase study; the first phase will consist in developing the intervention using a user-centred design approach, and the second phase is an open multicentre parallel-group superiority randomized trial. The study will be conducted in two stroke units in France. For qualitative study, semi-structured interviews will be conducted with patients, caregivers and the case manager: between fifteen and twenty interviews will be planned depending on data saturation. This number is planned based on available literature on data saturation [22, 23], however, if new information emerges from the final interviews, additional interviews will be planned.

### Setting and patients

The target population is composed of adult acute stroke patients managed in the participating stroke units. In the Rhône county (1,859,524 inhabitants), two stroke units manage acute stroke patients: one stroke centre in the Lyon University neurological hospital, including 12 acute stroke beds, and one stroke unit in a general hospital in the north of department with 6 acute stroke beds. Stroke neurologists will recruit adult patients who have had a first confirmed ischemic or haemorrhagic stroke managed in the participating stroke units, living in the Rhône department, and whose discharge from the stroke unit directly home is planned. The study will include only patients presenting a modified Rankin score (m-RS) of 1 to 3 at discharge from the stroke unit. Patients presenting with aphasia may be included if an informal caregiver is willing to commit to participate in the follow-up with the case manager. Neurologists should inform patients and their caregivers and obtain their consent before their inclusion in the study. Patients already living in an institution prior to stroke, or unable to communicate in the case of absence of informal caregiver or unwillingness to participate in the follow-up will not be included.

### Randomization

Before discharge from hospital, patients will be randomly assigned to one of the 2 groups at a 1:1 ratio. Randomization will be stratified on centre and presence of a caregiver, and will be centrally allocated using the Ennov Clinical® software (version 8.2.0). Allocation sequence will generate by statistician.

**Fig. 1:**
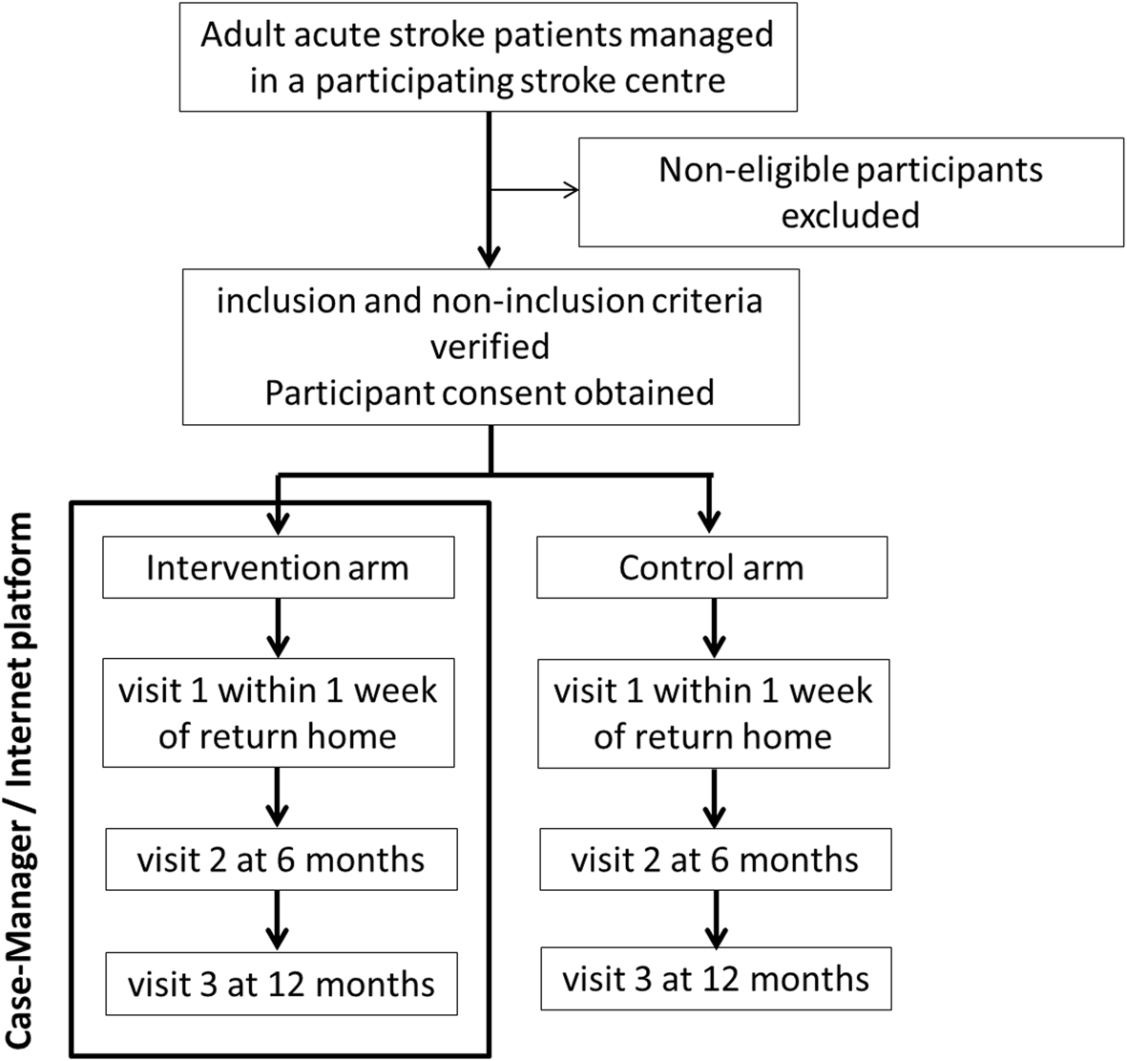
Flow chart of patients included in the NAVISTROKE trial

Figure 1 presents the study flowchart.

### Intervention

**Fig. 2:**
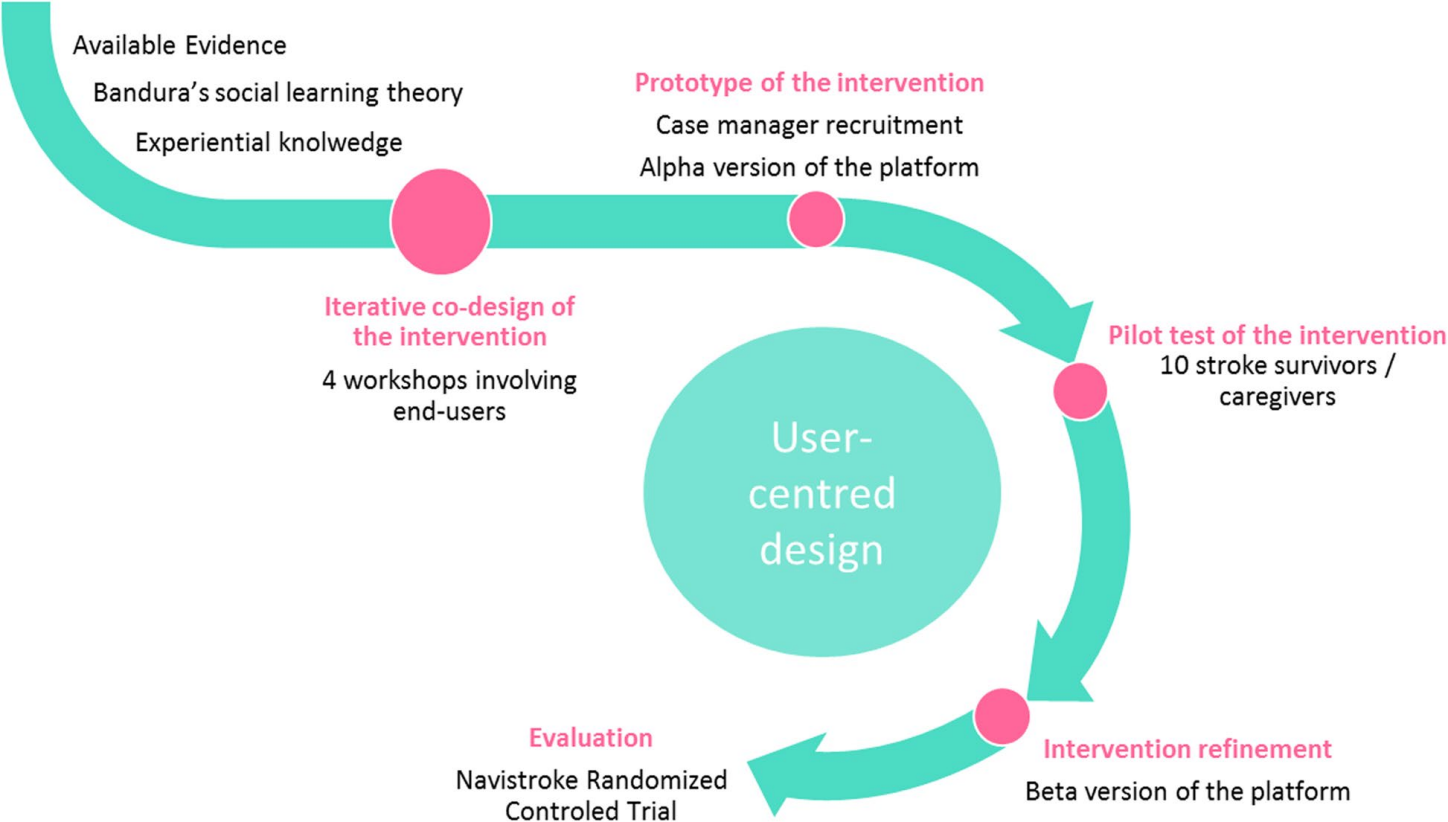
User-centred approach for the design of the intervention

**Fig. 3:**
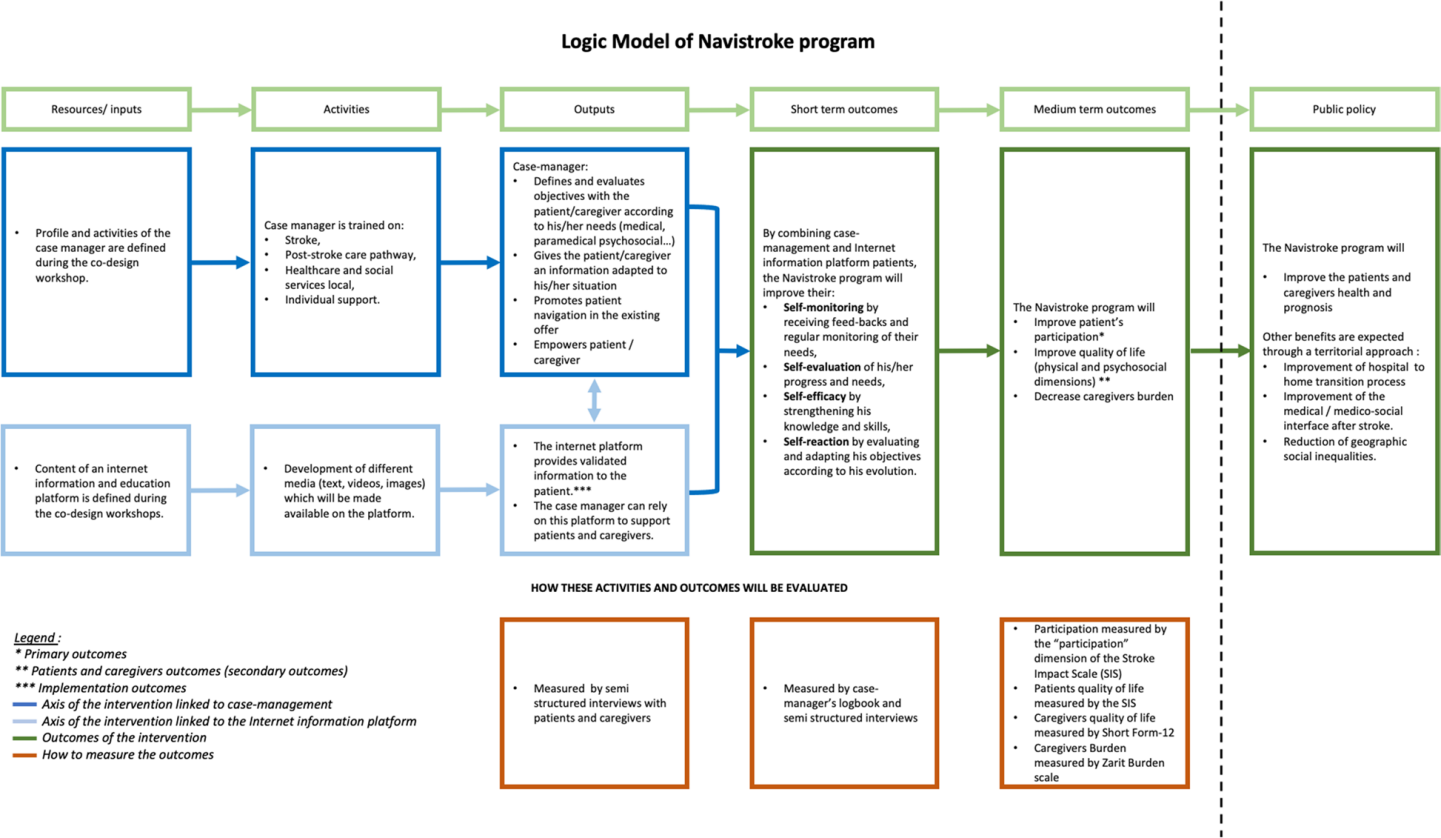
Logic Model of Navistroke program

Patients in the intervention group will receive telephone support by a trained case manager (number and frequency of contacts defined according to the patient’s needs) and access to an Internet information platform for a 12-month period after returning home following hospital discharge. The planned intervention is described in Table 1. It aims to improve patients’ ability to manage their situation and meet their needs upon returning home, including identifying and requesting the necessary health or social resources. The detailed intervention content will be codesigned during the first phase of the study, using a “user-centred design” approach [24] including the following steps: identification of end-user needs, prototyping/development of the intervention (case management procedures and platform), iterative improvement, end-users testing (Fig. 2). Four workshops involving end-users of the intervention will be planned. This will ensure that the intervention aligns with the context and integrates both experience from professionals’ skills and practices on the one hand, and patients’ and caregivers’ experiential knowledge on the other hand. The intervention content will be underpinned by the cognitive social theory [25, 26] and based on scientific literature, an overview of existing local organisations and the results from previous studies our team conducted on patient needs following the acute phase [27, 28]. The logic model of the intervention (case management + Internet information platform) making the link with the theory and expected outcomes is presented in Fig. 3. For the user-centred design approach, an advisory committee, composed of patients and caregivers, health professionals, social workers, and public health and social sciences researchers, will be formed. This committee will meet during 4 participatory co-design workshops facilitated by a social science researcher and will all last 2.5-3 hours. Following the grid described in Table 1, the advisory committee will: define the case manager’s profile, and required knowledge and skills; identify the resources and tools to be proposed on the Internet information platform; test the tools and content; refine the programme evaluation criteria; and test and validate the study procedures.

**Table 1:** Agenda of the co-design workshops during the user-centred design phase

**Workshop number**	**Topics covered**	**Objectives**	**Content and tools**	**Duration**
**Introduction**	Presentation of the project		Presentation of the project and participants	**30min**
	Setting the frame		Reminder of confidentiality and the role of each person (facilitators, peer-helper psychologist) Presentation (promotes participation and motivation)	
**Workshop 1**	The case manager Acknowledgements	Questioning the difficulties encountered during the patient care process Define the task profile of the case-manager	Presentation of the patient care pathway using a timeline Definition of the case manager Tools: PowerPoint slideshow drawing on social cognitive theory. The tasks and activities of the case manager and what is not part of his duties: Metaplan activity	**2h30min-3h**
**Workshop 2**	The case manager Resources Acknowledgements	Summary of Workshop 1 Define the general profile of the case manager Identify the necessary resources to be included on the web platform	Creation of a professional identity card listing the skills, knowledge, tasks, profile and training of the case manager Presentation of three ‘persona’ (platform user profiles) representing typic patient or caregiver portraits who could use the platform. Presentation of the platform by the graphic designer: brainstorming on strengths and weaknesses of the proposal identified by the participants (on content and form) Presentation of the web site architecture (different headings and contents) using a summary graph: brainstorming on strengths and weaknesses identified by the participants	**2h30min-3h**
**Workshop 3**	The web platform Acknowledgements	Summary of Workshop 2 Identify the necessary resources to be included on the web platform	Based on the information collected during workshop 2, presentation of version 1 of the platform by the graphic designer Gather feedback from participants. Strengths and limitations of the platform: Metaplan Adaptations envisaged by the participants	**2h30min-3h**
**Workshop 4**	The web platform Conclusion, Closing Acknowledgements	Summary of Workshop 3 Identify the necessary resources to be included on the web platform	Based on the information collected during workshop 3, presentation of version 2 of the platform by the graphic designer Gather feedback from participants. Strengths and limitations of the platform: Metaplan Adaptations envisaged by the participants	**2h30min-3h**

### Control (treatment-as-usual, TAU)

Patients in the control group will be followed-up according to usual practices that are not driven by the protocol or a structured process. Usually, the hospital discharge report is given to the patient at discharge and sent by postal mail to the general practitioner. Discharge prescriptions are explained to the patient and, if necessary, a social worker and/or occupational therapist may assist the patient or caregiver with administrative issues and preparation of home adaptations. After discharge, patient and caregiver support is based on the needs assessment made by the general practitioner and on patient and caregiver requests.

### Measurement of patient outcomes

The outcomes and data collection calendar are presented in Table 2 and linked with the intervention components in Fig. 3.

**Table 2:** Study schedule

		Patient			Caregiver			Instrument
Timing	Inclusion	1 week	6 months	12 months	1 week	6 months	12 months	
Baseline information	x							
PRIMARY OUTCOME								
Participation			x					The Stroke Impact Scale
SECONDARY OUTCOMES								
Participation				x				The Stroke Impact Scale
Quality of life		x	x	x	x	x	x	The Stroke Impact Scale and Short Form-12
Anxiety and depression		x	x	x	x	x	x	The Hospital Anxiety and Depression scale
Fatigue, sleep quality and sleepiness		x	x	x				The Pichot scale, the Pittsburgh scale and the Epworth scale
Zarit burden scale						x	x	The Zarit burden scale
PROGNOSIS OUTCOMES								
Stroke recurrence				x				Reported by the patient and/or caregiver and validated by checking the hospital discharge report
Death				x				
Rankin				x				The modified Rankin Score
Cognitive disorders				x				The Montreal Cognitive Assessment scale
ACCESS TO CARE OUTCOMES								
Consultations and hospitalizations		x	x	x				Reported by the patient and/or caregiver and a copy of medical prescriptions will be collected
Maintaining hospital discharge prescriptions		x	x	x				
Therapeutic persistence		x	x	x				
Occupational status				x			x	Reported by the patient and/or caregiver
Social isolation		x	x	x				The Social Support score Questionnaire
Patient activation Measure		x	x	x				The Patient activation Measure
Maintenance at home or institutionalization				x				Reported by the patient and/or caregiver
Satisfaction with the support received upon return home				x			x	Reported by the patient and caregiver through a structured questionnaire
Feeling of information about stroke and medical and social care			x	x		x	x	

#### Primary outcome

Patients’ participation at 6 months measured by the score obtained in the “participation” dimension of the stroke-specific quality of life scale: SIS 6 months after discharged home [29]. Participation is defined by the International Classification of Functioning, Disability and Health, as an individual’s involvement in life situations in relation to health conditions, body functions or structures, activities, and contextual factors [30]. Participation restrictions are problems an individual may have to fulfil his or her roles and engage in meaningful activities. SIS licence will be obtained before the first inclusion.

#### Secondary outcomes

Participation at 12 months: participation score at 12 months after discharged home (SIS). Quality of life at 6 and 12 months: scores of the other dimensions of the SIS at 6 and 12 months: force dimension, manual function, activities of daily living/instrumental activities of daily living (ADL/IADL), mobility, communication, emotions, memory/thinking, as well as global recovery.

Anxiety and depression scores: change of anxiety and depression scores between discharge home and 6 months, and discharge home and 12 months, measured by the Hospital Anxiety and Depression Scale (HADS) score [31]. Fatigue, sleep quality, and sleepiness: changes in fatigue level measured by the Pichot scale [32], sleep quality measured by the Pittsburgh scale, and sleepiness level measured by the Epworth scale between discharge home and 6 months, and discharge home and 12 months. Epworth scale licence will be obtained before the first inclusion.

Prognosis at 12 months after discharge home: stroke recurrence within 12 months, reported by the patient and/or caregiver and validated by checking the hospital discharge report; unscheduled hospitalisations or emergency room visits within 12 months of discharge from hospital to home reported by the patient and/or caregiver and validated by checking the hospital discharge report; m-RS at 12 months; death at 12 months reported by the caregiver; cognitive disorders at discharge to home and at 12 months, measured by the Montreal Cognitive Assessment (MOCA) scale [33, 34].

Prevention of cardiovascular risk factors: last known blood pressure, LDLc, and glycaemia values, as well as smoking status, physical activity, and body mass index at baseline, 6, and 12 months; these data will be collected by interviewing the patient or in the electronic medical record.

Access to care and social services at 12 months: consumption of care (consultations and hospitalisations) and perceived social aids; these data will be collected by interviewing the patient.

Maintain of the hospital discharge prescriptions at 6 and 12 months (persistence): 6 and 12 months maintenance of secondary treatments for stroke prescribed at discharge, and reasons for discontinuation if applicable; these data will be collected by interviewing the patient and a copy of medical prescriptions will be collected.

Occupational status at 12 months: return to work will be defined by working at least one day per week (10% full-time employment), and occupational status will be characterised as either resumption of the same professional activity, professional reclassification, adapted working time, or invalidity / early retirement.

Social isolation: social isolation at discharge, and at 6 and 12 months thereafter measured by the Social Support Questionnaire 6 [35]

Patient activation: Patient Activation Measure (PAM-13) score at discharge, 6, and 12 months [36]. PAM-13 licence will be obtained before the first inclusion.

Maintenance at home or institutionalization at 12 months.

Satisfaction with the support received upon return home, measured at 12 months by a structured satisfaction questionnaire developed for the study (Additional file 1).

Feeling informed: Feeling informed about stroke and medical and social care at 6 and 12 months through a structured questionnaire investigating perception on quality, understandability, and relevance of information provided by healthcare professionals (Additional file 1).

### Measurement of caregiver outcomes

Quality of life: change of the quality of life between discharge home and 6 months, and discharge home and 12 months, measured by Short Form-12 at discharged from hospital to home, 6 and 12 months [37].

Burden: change in burden measured by the Zarit burden scale at 6 and 12 months [38]. Zarit burden inventory licence will be obtained before the first inclusion.

Anxiety and depression scores: change in anxiety and depression scores between discharge home and 6 months, and discharge home and 12 months, measured by the HADS score.

Occupational status: change in professional activity since the patient’s return home (cessation of all professional activity, reduction in working time) in relation to the caregiving situation. Decrease in social and leisure activities since the patient’s return home in relation to the caregiving situation. Satisfaction with the support received upon return home and with information received, measured at 12 months by an ad-hoc questionnair

### Implementation process measurement

Context: elements of the context, external to the intervention, which may have modified the implementation or effect of the intervention. This will be studied using quantitative and qualitative approaches. Quantitative analysis will assess whether the efficacy of intervention differs according to the centre (stroke centre/stroke unit, usual organization of discharge in the unit), patient’s place of residence (rural/urban area), and healthcare density around the patient’s residence. External barriers and facilitators as perceived by the patients, caregivers and the case manager will also be collected during the semi-structured interviews.

Implementation: population reached by the intervention (characteristics of the patients who received the intervention, comparison with the target population), fidelity of intervention compared to the intervention that was planned, including case manager actions collected by the case manager in a logbook, and the use of the Internet information platform by patient (number of connections, duration of use, type of pages consulted). Any adaptations to the intervention will be collected in the semi-structured interviews with patients, caregivers as well as the case manager. Fidelity will be classified into 3 categories: implemented as planned, implemented with adaptations, not implemented.

Impact mechanisms: acceptability by patients, case managers and stroke professionals, strengths and limitations of the intervention, ownership and use of the intervention by patients and their caregivers, unexpected consequences and difficulties encountered by the case manager. These data will be collected by standardized questionnaires and semi-structured interviews among patients, caregivers and the case manager.

### Data collection

Data will be collected from the stroke unit medical charts by external research assistants (from the public health department). For each patient included in the trial, a case report form will be completed with his/her characteristics (age, sex, medical history, comorbidities, treatment, diagnosis, initial stroke severity as assessed by the National Institutes of Health Stroke Scale (NIHSS) at admission, pre-stroke autonomy as assessed by the pre-stroke m-RS).

Patients will be contacted within one week of discharge to home, as well as 6 months and 12 months thereafter for the collection of outcomes. This will be done by telephone [39] by trained research assistants of the study coordinating centre. Research assistants are experienced in stroke patient follow-up by telephone using the standardised scales used for this study. However, training by a stroke neurologist will be conducted before study implementation to ensure standardisation of data collection. Prior to each call, patients will receive the questionnaires by postal or email (or by hand at discharge) so that they can be read and pre-populate the questionnaires prior to the call. Patients will be given the choice of postal or email at inclusion. The data collected are listed in Table 2.

We will precisely detail support received by patients in the control group to describe the usual practices during the study.

### Data management, confidentiality, and dissemination

All information required by the protocol will be recorded in an electronic case report form (eCRF). This eCRF, specific to the study, will be developed by a data manager from the Hospices Civils de Lyon on the Ennov Clinical^®^ software (version 8.2.0).

The data set will be computerised in a coded way, in accordance with the law for data protection and freedom of information (Article L.1121-3 of the French Public Health Code). The study patients will be identified by a unique study inclusion number and by the first initial of their surname and of their given name.

Data should be entered, as soon as they are collected, by the authorised persons (investigator and personnel recorded on the delegation log) and having at their disposal their own login name according to the law for data protection and freedom of information.

Access to the data will be restricted to only the persons participating in the study. Authentication will be made using passwords, which will be regularly changed. The investigators and clinical research assistants of an investigating centre will only have access to the data for their patients and will enter the data directly into the eCRF using a secured website. The investigator will be responsible for the reliability of the data entered and must complete the data as it is obtained during the patient’s follow-up. Throughout the length of the study, the data will be stored in an ISO 27001-certified data centre and backed up daily.

### Sample size

The study will be powered to detect a difference of 15 points in the SIS-Participation score at 6 months of follow-up between the TAU and the intervention group. A 15-point difference between groups in SIS-P score is considered clinically relevant [40]. To detect this difference, 70 patients per group is needed (assuming a common SD of 27 according previous results from the STROKE-69 cohort, power=90%, bilateral alpha level=0.05). Accounting for 20% attrition, we plan to recruit a total of 170 patients.

### Recruitment

The annual number of stays of eligible patients recorded in the two participating centres guarantees the feasibility of this recruitment. In a previous cohort study conducted in these two centres, over 12 months a total of 612 acute strokes were treated in the stroke center and 208 in the stroke unit [28]. Considering that 70% of these patients return directly to their homes following the acute phase, the number of potentially eligible patients would be 574 over 12 months. Recruitment of 170 patients over 12 months, or 15 patients per month, is therefore feasible; this rate of inclusion is also consistent with the case manager’s follow-up who would include 7 to 8 new patients in the intervention group per month over 12 months.

### Patient and Public Involvement

The NAVISTROKE study will be conducted in partnership with patient representatives, using different approaches and these at several steps of the study. Patient participation in the study is underpinned by the patient engagement continuum [41, 42]. Two patients from the local stroke patient association (France AVC69) are involved as partners in the research since the first step of the study before grant submission. These two patients are not trained in the science of partnership in research but they have a strong experience of collaboration over several years with our team of researchers and clinicians. They are full members of the study steering committee, and, as such will be involved in decisions throughout the study, in study conduct, in the interpretation of results, and in knowledge transfer activities. The only task they will not be involved in is the recruitment of study participants.

We will also involve a sample of patients (5 to 10) in an advisory committee during the user-centred design phase to define the content of the intervention. Patients will participate in workshops with professionals to give their perspectives regarding the intervention. They will be recruited among volunteers from participants of a previous stroke cohort study [28].

In order to optimize participant engagement in the workshops, we designed a recruitment protocol based on the theory of engagement in social psychology [43] and on the patient partner recruitment guide of the University of Montreal [44]. This protocol consists of four steps: pre-selection by the clinical team; telephone interview; face-to-face interview.

This will allow us to rigorously prepare and inform patients so that the codesign workshops can take place in the best possible way.

### Statistical analysis

Categorical variables will be presented using numbers and percentages, and quantitative variables using mean and standard deviation or median and interquartile range according to their distribution.

Group difference in the SIS Participation domain score at 6 months after hospital discharge will be analysed using a linear model adjusted on the stratification factors (centre and presence or caregiver). The primary analysis will be performed in accordance with the intention-to-treat principle (an imputation method for missing data will be used when appropriate). In a modified intention-to-treat analysis and per-protocol analysis, patients who died before 6 months or those with stroke recurrence before 6 months will be excluded. Change in the SIS participation domain score between 6 and 12 month after discharge will be compared between groups using linear mixed effects model. The model will include patients as random effect, time (6 and 12 months), group (TAU or intervention) and an interaction effect of treatment group × time as fixed effects after controlling for the stratification factors.

Continuous secondary outcomes will be assessed in a similar way. Categorical data will be analysed using logistic or survival models. For non-repeated continuous and binary measurements, ordinary linear regression and logistic or Cox proportional-hazards models will be used when appropriate.

A detailed statistical analysis plan will be made prior to database lock. Analysis and reporting of the results will follow the CONSORT guidelines for reporting randomised controlled trials [45]. Analyses will be conducted using SAS software (version 9.4; SAS Institute Inc., Cary, NC, US). All tests will be two-sided and carried out at the 5% level of significance.

### Qualitative study

During the 12-month visit, clinical research assistants will propose to a sample of patients and their informal caregiver from the intervention group to participate in individual semi-structured interviews. These interviews will help better understanding the processes of effect of the intervention and the strengths and limitations of the transition programme. The interviews will take place preferentially at the individuals’ homes, with the patient alone and with the caregiver alone. They will be conducted by a social psychologist and based on an interview guide elaborated based on Bandura’s theory [25, 26] and validated by the steering committee. An interview will also be carried out after the last patient’s follow-up with the case manager using a specific interview guide (Additional file 2).

The interviews will be audio-recorded and transcribed for analysis. The analysis will focus on data from verbatim, interview notes, and the case manager’s logbook data. A thematic analysis of the content following the approach proposed by Bardin [46] will be carried out using NVIVO software (Nvivo QSR International). A vertical and transversal analysis will be carried out to categorise the verbatim into themes and sub-themes. The analysis grid will follow the themes of the interview grid, enriched during the analysis of emerging themes and sub-themes. The different data sources (interviews, notes, and logbooks) and populations (patients, caregivers, case managers) will be triangulated. The results will then be combined with the results of the quantitative data and collected to analyse the implementation.

### End of study visit

The end of the search for patients will take place at the end of the 12-month visit. End-of-study data will be collected by telephone by a clinical research assistant; the data collected is specified above.

For patients participating in the qualitative study, participation in the interview will mark the end of the study.

Given the nature of the intervention and the low risk of serious research-related adverse events, no independent monitoring committee is planned for this study

Only the statistician and the coordinating centre will have access to the final trial dataset.

## Discussion

Available evidence shows that the complex needs of stroke patients and caregivers are not being met upon returning home after hospital discharge [4–6]. According to our previous research and international data, a large number of patients face difficulty identifying and receiving the health and social services they would need after returning home [5]. They frequently find themselves isolated and out of care, which has significant negative consequences on prognosis [5, 6]. This led us to develop a patient-centred programme that will, through individualised and tailored follow-up, help the patient and their caregiver to better identify, understand, and use the care and services available. The programme will promote the activation and participation of the patient and/or their caregiver, reduce care disruptions by improving the hospital/ambulatory transition, and the medical/medico-social interface. The final goal of this program is to support stroke survivors and their relatives to engage meaningfully in their life activities, based on their needs and personal objectives.

The implementation of a support programme adapted to the needs of the patients and their environment also aims to reduce social inequalities of health. Indeed, people who are the most affected by the lack of information and ability to know and use the existing arrangements are those with a lower level of education [5, 6]. The intervention will take into account low literacy levels and disabilities that might impair access to and use of health information. Tailoring of the information will be ensured by the association of the two components, Internet information platform and case manager follow-up. The case manager will be trained in communication and in interaction with low-literacy patients [47, 48]. The intervention model including a centralised case manager, with contact by telephone, and the online platform will also make it possible to disseminate this intervention over a wide area and even in case of geographical isolation. Patient partnership in the study will also help design an intervention that does not lead to foster inequalities.

The study has, however, limitations. Indeed, the evaluation of the efficacy of the intervention will be carried out in a single geographic area. However, this area is covered by 2 stroke unit, including one comprehensive stroke centre and one primary stroke centre. The stratification of the randomisation on the centre will make it possible to analyse the centre effect and the process evaluation will help in understanding any difference in the implementation of the intervention that might be due to centre organisation. These data will help to better define optimal conditions to further scale the intervention to a larger dimension.

Strengths of the study are the patient-centred approach engaging patient partners throughout the study, the flexible, individualised, theory-informed and evidence-based content of the programme, supported by existing organisations and easily generalizable to a national scale. The mixed method design, associating a two-centre prospective randomized trial and semi-structured interviews with patients, caregivers and the case managers, also ensures a high level of evidence and an in-depth understanding of the impacts of the intervention.

This project may contribute to improve post-stroke recovery and improve patient’s quality of life. Afterwards, this innovative comprehensive and patient-centred transition programme may be transferred to other settings or to patients suffering from other sudden disabilities such as head injuries.

## Supplementary Information

**Additional file 1.** Questionnaire developed for measure satisfaction with the support received upon return home and feeling of information about stroke and medical and social care

**Additional file 2.** Interview guide *specific interview guide for patients, caregivers and case manager

## Data Availability

Data will be analysed after completion of the study, they will be available from the corresponding author upon reasonable request. A scientific paper will be published in a peer-reviewed ICMJE journal and a written communication will be sent to the study participants to present the main results. Only the full protocol may be made available to the public upon request to the corresponding author.

## Abbreviations

ADL: Activities of daily living
eCRF: Electronic case report form
HADS: Hospital Anxiety and Depression Scale
IADL: Instrumental activities of daily living
MOCA: Montreal Cognitive Assessment
m-RS: Modified Rankin score
NIHSS: National Institutes of Health Stroke Scale
SIS: Stroke Impact Scale
