# Supplementary material for: Co-design and evaluation of a patient-centred transition programme for stroke patients, combining case management and access to an internet information platform: study protocol for a randomized controlled trial - NAVISTROKE
Source: BMC Health Serv Res. 2022 Apr 22;22:537. doi: 10.1186/s12913-022-07907-5 (PMC9027042; doi:10.1186/s12913-022-07907-5)
Supplement: Supplementary file 1 — Additional file 1. Questionnaire developed for measure satisfaction with the support received upon return home and feeling of information about stroke and medical and social care [file 12913_2022_7907_MOESM1_ESM.docx]

**Additional file 1: Questionnaire developed for measure satisfaction with the support received upon return home and feeling of information about stroke and medical and social care**

**Perceived satisfaction with the support received at discharge**

On a scale of 0 to 10, where 10 means maximum satisfaction and 0 means no satisfaction, how satisfied were you with the support you received during the discharge transition ? (please circle your measure)

| Very low | Moderate | Very high |
| --- | --- | --- |
| 0------1------2------3------4------5------6------7------8------9------10 | | |

On a scale of 0 to 10, where 10 means maximum satisfaction and 0 means no satisfaction, how satisfied were you with the support you received after discharge? (please circle your measure)

| Very low | Moderate | Very high |
| --- | --- | --- |
| 0------1------2------3------4------5------6------7------8------9------10 | | |

Have you (or your relatives) been involved in decisions about your discharge as much as you wanted?  YES  NO

Did you (or your relatives) feel able to participate in decisions about your return home?  YES  NO

Did you (or your relatives) feel comfortable to ask all questions about your return home?  YES  NO

Did you receive an attentive ear from health professionals to ask questions about your discharge?

- Never
- Rarely
- Sometimes
- Often
- Always

What do you think of the way your discharge was organised at hospital (announcement of your discharge date, planification, continuity of care, home reorganisation...)?

- Bad
- Low
- Average
- Good
- Excellent

During the transition process (including hospital and outpatient services):

Did you (or your relatives) feel that you received sufficient support to get the services you needed at home?

YES  NO

Did you (or your relatives) feel that you received sufficient support to get the equipment you needed at home?

YES  NO

Did you (or your relatives) know whom to contact if you had problems relating to you stroke or rehabilitation?

YES  NO

Did you (or your relatives) receive sufficient emotional support after discharge?

YES  NO

**Feeling of information**

1/ During your hospital stay:

Did you receive during hospitalization spontaneous explanations by health professionals (without asking) about your health condition, treatment, care, etc.?

- Never
- Rarely
- Sometimes
- Often
- Always

Did you (or your relatives) feel that you had been given all the information needed to prepare your discharge?

YES  NO

Did you (or your relatives) feel that you had been given all the information needed to understand your condition (recovery and rehabilitation)?

YES  NO

2/ Since you left the hospital,

Did you (or your relatives) feel that you had been given all the information needed to understand your condition (recovery and rehabilitation)?

YES  NO

Did you (or your relatives) knew where or from whom getting the information needed regarding your condition (recovery and rehabilitation)?

- Never
- Rarely
- Sometimes
- Often
- Always

Where or from whom did you get information about your stroke and rehabilitation?

- Hospital professionals
- General practitioner
- Nurse or home professional caregivers
- Patients associations, peers (other stroke patients)
- Family or relatives
- Leaflets or brochures
- Internet, mobile apps
- Other  please specify __________________________

How did you find the information you get about your medical care?

- Sufficient  YES  NO
- Reliable  YES  NO
- Relevant  YES  NO
- Understandable  YES  NO

How did you find the information you get about your home organization?

- Sufficient  YES  NO
- Reliable  YES  NO
- Relevant  YES  NO
- Understandable  YES  NO

How did you find the information you get about your navigation of the health system (finding services and equipment needed)?

- Sufficient  YES  NO
- Reliable  YES  NO
- Relevant  YES  NO
- Understandable  YES  NO

How did you find the information you get about the social offers?

- Sufficient  YES  NO
- Reliable  YES  NO
- Relevant  YES  NO
- Understandable  YES  NO

How did you find the information you get about emotional support?

- Sufficient  YES  NO
- Reliable  YES  NO
- Relevant  YES  NO
- Understandable  YES  NO
