# Supplementary material for: Co-design and evaluation of a patient-centred transition programme for stroke patients, combining case management and access to an internet information platform: study protocol for a randomized controlled trial - NAVISTROKE
Source: BMC Health Serv Res. 2022 Apr 22;22:537. doi: 10.1186/s12913-022-07907-5 (PMC9027042; doi:10.1186/s12913-022-07907-5)
Supplement: Supplementary file 2 — Additional file 2. Interview guide *specific interview guide for patients, caregivers and case manager [file 12913_2022_7907_MOESM2_ESM.docx]

**Additional file 2: interview guide**

| **CASE MANAGER** | | |
| --- | --- | --- |
| **Themes** | **Questions** | **Reminders** |
| **1/ intervention with patients and caregivers** | Can you tell me about the different tasks and activities you have carried out?  What skills did you use during your interventions? | - for patients - for caregivers - Banduras theory and logic model of the intervention: goal setting, monitoring and feedback, empowerment |
| **2/ link with patients and caregivers** | How would you describe the relation you have created with patients? with caregivers? | - Easy or difficult to create? Issues and challenges - Evolution over time, adjustment |
| **3/ collaboration with the health care system** | How did you collaborate with the stroke unit?  How did you take into account existing arrangements in your interventions? | - Professional recognition (listened to and supported by the care team/difficulty of being alone) |
| **4/ Job experience/satisfaction** | How did you feel in this new role of case manager?  How do you see the implementation of this new profession in the field of stroke?  How satisfied are you with this new role? | - Did you encounter any difficulties during your interventions? - How were they overcome? - Did you ask for external help? |

| **PATIENTS AND CAREGIVERS** | | |
| --- | --- | --- |
| **Themes** | **Questions** | **Reminders** |
| **1/ Case manager** | How, when and why did you meet or call the case-manager?  What were the benefits you perceived from the case manager follow-up? | - Frequency of use - What assistance was provided? - Self monitoring, self-evaluation, self efficacy, self-reaction, empowerment - Satisfaction |
| **2/ Internet information platform** | How, when and why did you use the platform?  How did the Internet information platform help you? | - Frequency of use - Ease of use - Inputs - Self monitoring, self-evaluation, self efficacy, self-reaction, empowerment |
| **3/ Meeting needs and expectations** | How have the arrangements in place met your needs/expectations? | - Adaptation to the personal situation and its evolution - Impact on daily life - Links with other health or social services |
| **4/ Limitations and improvements** | How could we improve the program, what improvements could/should be made ? | - What was missing/should be added - What is surplus/should be removed (was not useful, should not be part of the devices...) |
